# Simultaneous occurrence of cerebellar medulloblastoma and pituitary adenoma: A case report

**DOI:** 10.1186/1757-1626-1-175

**Published:** 2008-09-23

**Authors:** Vassilis Samaras, Efstathios Samaras, Ioanna Stergiou, Paraskevi Konstantopoulou, Christos Arnaoutoglou, Marianthi Arnaoutoglou, Vassilis Varsos, Calypso Barbatis

**Affiliations:** 1 Department of Pathology, Hellenic Red Cross Hospital, Athens, Greece; 2 Department of Neurosurgery, Hellenic Red Cross Hospital, Athens, Greece; 3 Department of Radiology, General Hospital of Lamia, Lamia, Greece; 4 Department of Cytopathology, Evangelismos General Hospital, Athens, Greece; 5 1st Department of Neurology, Aristotle University of Thessaloniki, Thessaloniki, Greece

## Abstract

**Purpose:**

We present the unusual occurrence of two distinct neoplasms in a 42-year-old woman with an operated pituitary adenoma 18 years ago.

**Methods:**

Clinical history, magnetic resonance imaging studies and histopathological findings were utilized for our diagnostic considerations.

**Results:**

Concomitant presence of a cerebellar medulloblastoma secondary disseminated within the spinal canal and a pituitary macroadenoma, was identified.

**Conclusion:**

To the best of our knowledge, this is the first reported case in which these two neoplasms are simultaneously occurred in the same individual. A short review of the literature is performed.

## Introduction

Medulloblastomas are posterior fossa tumors preferentially manifested in children less than 15 years and rarely occurring in adults [1]. These tumors, belonging to the family of central primitive neuroectodermal tumors (cPNETs), are highly malignant entities with an inherent tendency to metastasize via the cerebrospinal fluid (CSF) pathway [1]. This phemomenon occurs in up to 30% of all patients at the time of initial diagnosis [1,2] with some researchers believing that all patients have already metastatic disease in the spinal cord at the time of first detection of the tumor [2].

Pituitary tumors constitute about 10–15% of all intracranial tumors [3]. Pituitary adenomas, in particular, belong to the family of pituitary tumors and they are defined as benign neoplasms located in the sellar turcica [3]. The treatment, consisting of surgery, irradiation and pharmaceutical therapy or a combination of them, targets at the control of tumor cell proliferation and the reduction of hormone secretion (in the case of endocrine active neoplasms) [4,5]. These tumors, however, carry a risk for recurrence even many years after the initial treatment [3,5].

Simultaneous occurrence of two different tumors in a patient, including cases which concern the central nervous system (CNS), has been previously described in the literature [6-9]. This rare phenomenon is mainly associated with cases in which two concurrent but histologically unrelated neoplastic elements have collided with each other, in the same organ [8].

In this context, we report the case of a 42-year-old woman suffering simultaneously from a cerebellar medulloblastoma, secondary disseminated within the spinal canal, and a pituitary macroadenoma. The patient had been operated 18 years before due to a pituitary adenoma. Taking into account the published data, for the first time a medulloblastoma coexists with a pituitary tumor.

## Case presentation

A 42-year-old woman was admitted to the Hellenic Red Cross Hospital with diminished function of her right leg as well as sensory disturbances of one week duration. Neurological examination revealed paresis of the right leg, and increased tendinous reflexes in both legs. The abdominal reflexes were all abrogated. During hospitalization the patient showed deterioration of neurological symptoms with severe paresis of both legs as well as urinary incontinence.

The specific details of patient's history were as follows: Occupation: housewife; Ethnicity: Ukranian; Weight: 65 Kgr; Height: 180 cm; Medical history: previous surgery 18 years ago due to a pituitary adenoma-no data available regarding the completeness of the resection and the administration or not of irradiation therapy; Family history: unremarkable; Pregnancies: no; Smoking: no; Drinking alcohol: no; Current medications: no.

**Figure 1:**
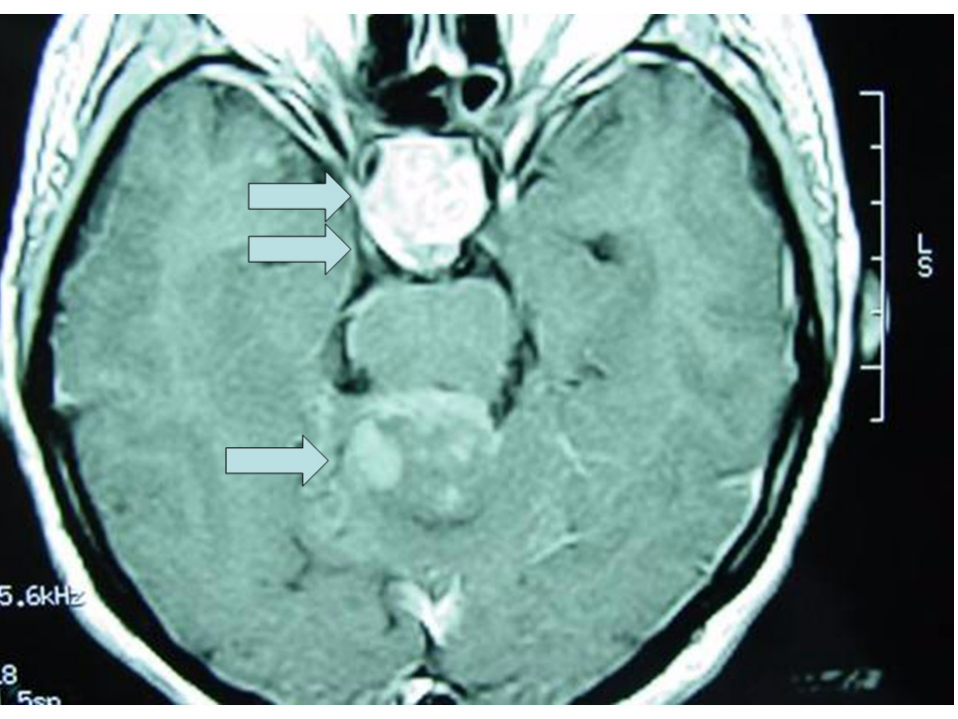
T1-weighted MRI: Tumor of the posterior cranial fossa (single arrow) as well as the macroadenoma of the pituitary gland (double arrow).

Magnetic resonance imaging (MRI) of the brain demonstrated an inhomogeneously contrast-enhancing, multilobulated tumor of the posterior cranial fossa, measuring 3 × 3 × 2,3 cm [Fig. 1]. The mass was hyperintense in T2-weighted images and hypointense in T1-weighted images relative to cortical grey matter. The tumor had an intimate liaison with the cerebellum surrounding the upper level of vermis and cerebellar hemispheres and projecting into the roof of the fourth ventricle. The mass caused a compressive effect into the posterior surface of the brainstem's peduncles, displacing the aqueduct of Sylvious. It was not observed supratentorial extension of the lesion, the epiphysis was normal, the ventricles were not dilated and it was not noted any subdural or epidural blood collection.

The imaging studies, additionally, showed a recurrent macroadenoma of the pituitary gland with greatest diameter 2,8 cm [Fig. 1]. The mass caused expansion of the sellar turcica, occupying the suprasellar cisterns, mainly on the right. Furthermore, the tumor compressed the optical chiasm and the ipsilateral optical fibers, especially on the right. Compressive results were also observed on the third ventricle as well as on the hypothalamus. The mass was characterized from heterogeneous, intense uptake of the contrast material and was closely related to carotid siphons, mostly on the right. Biochemical tests, however, did not show any abnormality regarding the levels of the pituitary hormones.

**Figure 2:**
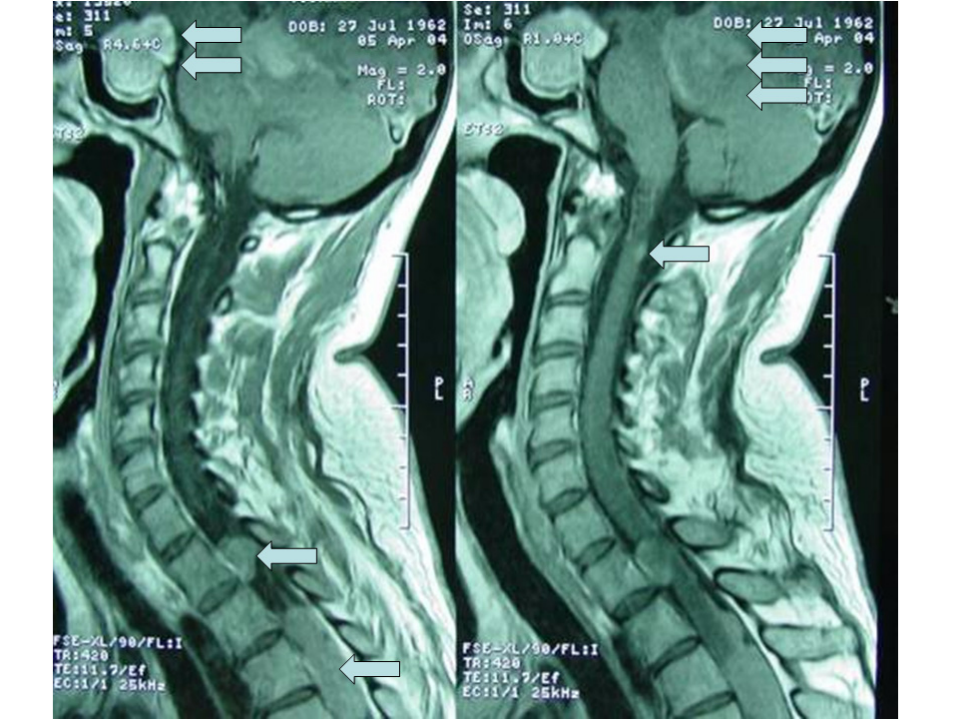
T1-weighted MRI: Tumors of the cervical and thoracic cord (single arrow). In addition, the tumors of the sellar turcica (double arrow) and cerebellum (three arrows) are depicted.

MRI of the spinal cord, also, showed multiple intradural-extramedullary masses with obstructive effects especially in the upper level of thoracic spinal cord as well as small-size tumors in the entire length of the spinal cord. More specifically, the contrast-enhancing masses of the upper thoracic cord had greatest diameter from <10 mm to 3,2 cm and were isointense in T1 and T2-weighted images relative to spinal cord [Fig. 2]. The biggest tumor was accompanied by small satellite lesions and encased the spinal nerve roots displacing some of them laterally. Surgical intervention took place in order to decompress the thoracic cord in the level of T3–T7. Nevertheless, the operation had only a debulking result, since the tumor was intimately connected with a large number of nerve roots.

A non-circumscribed, soft, friable, partly necrotic mass, measuring 3 × 1,5 × 0,5 cm, from the biggest mass in the thoracic spinal cord, was excised, fixed in formalin 10% and embedded in paraffin. The histological and immunohistochemical profile of multiple tissue sections was analyzed using the conventional stain with hematoxylin/eosin and the Bond-maX system (Vision BioSystems Ltd, Australia) according to the manufacturer's instructions. The Primo Star upright microscope and the Axio Cam IC camera (Carl Zeiss MicroImaging Inc, North America) were also utilized for assessing and capturing of images.

**Figure 3:**
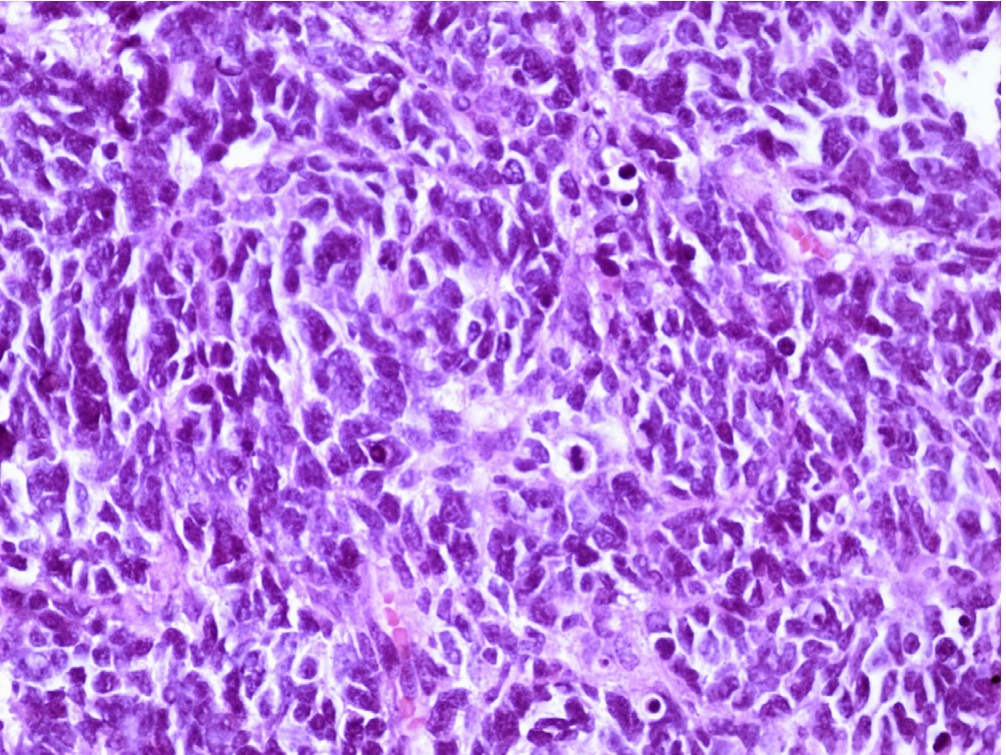
Hematoxylin/Eosin (×400 magnification): Surgically excised lesion of the thoracic spinal cord: Solid, small cell malignant neoplasm with ill-defined rosettes.

Specifically, a small cell malignant tumor with ill-defined rosettes was identified [Fig. 3]. Immunohistochemical analysis demonstrated that the neoplastic cells were diffusely positive for NCAM and Vimentin and focally for Synaptophysin, NSE and C-Kit. GFAP staining was only positive in reactive astrocytes. MCK, S-100, LCA, CD99, Desmin, Chromogranin-A and Myo-1 were all negative. The Ki67 index was >50%.

These histopathological results along with the MRI findings of the brain's examination were consistent with a diagnosis of a central PNET (medulloblastoma) of the cerebellum with secondary dissemination through the CSF.

The mass of the cerebellum was not excised as it did not demonstrate serious compressive results and it was necessary to avoid any further aggravation of the general medical status of the patient. The patient received CNS irradiation in another medical center but chemotherapy was not administered since the patient refused any further medical treatment. Unfortunately, the patient died one year after the initial diagnosis.

## Discussion

We described the case of a medulloblastoma primary localized in the cerebellum of a 42-year-old woman with secondary dissemination through the CSF pathway. However, our case was further complicated as it was detected a mass in the sellar region with an imaging appearance fully consistent with a pituitary macroadenoma. To the best of our knowledge, this is the first report in the literature of a medulloblastoma coexisting with a pituitary tumor, in the same individual.

Regarding differential diagnosis, a pituitary carcinoma, developed in the ground of the adenoma and exhibited metastatic lesions in the CNS, including the cerebellum as well as the spinal cord, was the main entity under consideration. The histological features, however, as well as the immunohistochemical profile (chromogranin A negative and vimentin positive) of the lesion of the spinal cord excluded this diagnosis. Furthermore, there was not noted any evidence of metastatic disease elsewhere, a finding usually observed in pituitary carcinomas [3]. Thus, according to the MRI findings and the clinical history, we deduced that the pituitary tumor was a recurrent pituitary adenoma, 18 years after the initial operation for the first pituitary tumor.

As a matter of fact, the occurrence of two distinct tumors within CNS has been previously described in the literature. Interestingly, Gironimi et al. in 1981 reported the case of a primary Burkitt-type lymphoma of the CNS, developed in an 11-year-old boy 6 months after extirpation of an astrocytoma, stressing the notion of collision tumors and their differential diagnosis [6]. Other researchers, additionally, have been described the coexistence of oligodendroglioma with anaplastic astrocytoma [9], pleomorphic xanthoastrocytoma [7], metastatic breast carcinoma [10] and juvenile pilocytic astrocytoma [11], within CNS.

Furthermore, Curto et al. recently presented the concurrent presence of pituitary adenoma, intracranial meningioma and cerebral aneurysm highlighting the suggestion that growth hormone or other growth factors could play a role in appearance or in growth of meningioma [12]. Previously, Cannavò et al. had reported the coexistence of an eosinophilic pituitary adenoma and an endotheliomatous meningioma, in the sellar region [13].

Attempting to explain the emergence of the medulloblastoma in our patient, two hypotheses could be considered. The first one is related to a possible histogenetic or clinical (as far as a specific syndrome is concerned) interconnection between medulloblastomas or cPNETs and pituitary adenomas. However, there is no bibliographical evidence for this kind of association and thus the above mentioned theory must be excluded.

On the other hand, according to the second assumption, the medulloblastoma could be developed as a result of the previous therapeutic manipulations for the pituitary tumor. Specifically, the patient most probably received irradiation for the initially presented pituitary adenoma, 18 years ago. Therefore, given that the occurrence of a second intracerebral neoplasm after craniospinal radiotherapy is well recognized [14,15], it could be suggested that this is the case regarding our patient.

Indeed, the estimated risk for the aforementioned second tumors is thought to be as high as 1–2%, occurring with a latency of 8–15 years [15]. A wide range of neoplasms can result after irradiation for any kind of CNS malignancy (including medulloblastomas), with a predominance of astrocytomas, ependymomas, multiple meningiomas, schwannomas as well as malignant gliomas and sarcomas [14,15].

## Conclusion

In summary, regardless of the various explanations which can be given, we reported the peculiar concomitant presence of a disseminated medulloblastoma and a pituitary macroadenoma. The former tumor most probably progressed in the context of a previous irradiation therapy for the initial pituitary adenoma, 18 years ago.

The major reason for which we consider irradiation as the most likely causative factor for the expansion of the new tumor is that radiotherapy accounts for the growth of a second tumor in many other instances [14]. It should be also emphasized that there is no proof of histogenetic or clinical association between pituitary adenoma and medulloblastoma, so far. However, we cannot exclude the possibility of a specific yet undescribed genomic imbalance which is responsible for the simultaneous progression of these two tumors. Further studies in a molecular level may elucidate this phenomenon.

## Consent

Written informed consent was obtained from the patient, at the time of initial diagnosis and prior surgery, for publication of this case report and accompanying images. A copy of the written consent is available for review by the Editor-in-Chief of this journal.

## Competing interests

The authors declare that they have no competing interests.

## Authors' contributions

VS: Writing of manuscript, histological diagnosis. ES: Writing of manuscript, acquisition of clinical data. IS: Conception and design of case report. PK: Analysis of imaging findings. CA: Analysis and interpretation of clinical data. MA: Analysis and interpretation of clinical data. VV: Revising of manuscript. CB: Revising and editing of manuscript, histological diagnosis. All authors read and approved the final manuscript.
